# Program *VUE*: analysing distributions of cryo-EM projections using uniform spherical grids

**DOI:** 10.1107/S1600576724002383

**Published:** 2024-05-10

**Authors:** Ludmila Urzhumtseva, Charles Barchet, Bruno P. Klaholz, Alexandre G. Urzhumtsev

**Affiliations:** a Architecture et Réactivité de l’ARN, UPR 9002 CNRS, IBMC, Université de Strasbourg, 15 rue R. Descartes, 67084 Strasbourg, France; b Centre for Integrative Biology (CBI), Department of Integrated Structural Biology, IGBMC (Institute of Genetics and of Molecular and Cellular Biology), Centre National de la Recherche Scientifique (CNRS) UMR 7104, Institut National de la Santé de la Recherche Médicale (Inserm) U964, Université de Strasbourg, 1 rue Laurent Fries, 67404 Illkirch, France; c Université de Lorraine, Physics Department, 54506 Vandoeuvre-lès-Nancy, France; DESY, Hamburg, Germany

**Keywords:** 3D reconstruction, cryo-EM, view frequency, preferred orientations, uniform grids, Lambert projection

## Abstract

Three-dimensional cryo electron microscopy reconstructions are obtained by extracting information from a large number of projections of the object. These projections correspond to different ‘views’ or ‘orientations’, *i.e.* directions in which these projections show the reconstructed object. Uneven distribution of these views and the presence of dominating preferred orientations may distort the reconstructed spatial images. This work describes the program *VUE* (views on uniform grids for cryo electron microscopy), designed to study such distributions. Its algorithms, based on uniform virtual grids on a sphere, allow an easy calculation and accurate quantitative analysis of the frequency distribution of the views. The key computational element is the Lambert azimuthal equal-area projection of a spherical uniform grid onto a disc. This projection keeps the surface area constant and represents the frequency distribution with no visual bias. Since it has multiple tunable parameters, the program is easily adaptable to individual needs, and to the features of a particular project or of the figure to be produced. It can help identify problems related to an uneven distribution of views. Optionally, it can modify the list of projections, distributing the views more uniformly. The program can also be used as a teaching tool.

## Abstract 2

The program *VUE* analyses the distribution of 2D projections in cryo-EM and presents this information in a quantitatively exact manner via maps and other diagrams.

## 1. Introduction

In cryo electron microscopy (cryo-EM), three-dimensional (3D) reconstructions are obtained from two-dimensional (2D) projections of the object. There are two priors to achieve correct results:

(i) The object is unique and structurally homogeneous, otherwise classifications and structure sorting are required.

(ii) The orientations of the object corresponding to the projections are evenly distributed in space, otherwise the frequency of occurrence of the same or close orientations should be considered.

Each 2D projection allows recovery of a section of the Fourier space [concerning Fourier sampling see, for example, Baldwin & Lyumkis (2021 ▸)]. In practice, projection values are available on a regular grid of some virtual unit cell. Therefore, the discrete Fourier transform (DFT) of these values allows the reconstruction of the plane crossing the origin (the central section) of the respective Fourier coefficients of the electrostatic scattering potential of the sample. Stated more accurately, the DFT provides a discrete finite set of approximations to these coefficients.

It is understandable theoretically, and has been observed in practice, that both under- and over-represented projections distort the reconstructed 3D image [see *e.g.* Tan *et al.* (2017 ▸) and Baldwin & Lyumkis (2020 ▸)]. The negative effects of different kinds of systematically missed Fourier coefficients have previously been practically demonstrated for crystallographic maps [see *e.g.* Lunin (1988 ▸), Lunin & Skovoroda (1991 ▸) and Urzhumtsev (1991 ▸)] and the respective software reported (Urzhumtseva & Urzhumtsev, 2011 ▸). According to Sorzano *et al.* (2021 ▸), ‘*The presence of preferred orientations in single-particle analysis (SPA) by cryo-electron microscopy is currently one of the hurdles preventing many structural analyses from yielding high-resolution structures.*’ Dealing with this problem requires answering several related questions:

(i) How to calculate the frequency of different projections, also known as orientations.

(ii) How to express this information, in particular that about over- and under-represented orientations, accurately and in an efficient way.

(iii) How to use this information to analyse possible deformations of the 3D reconstruction and eventually to improve the latter.

In this work, we address the two first questions. A practical answer to the third question is a separate project for which the tools discussed in this article can be used.

Historically, and most frequently, projections are characterized by various choices of Euler angles, *e.g.* the approaches used by van Heel & Keegstra (1981 ▸) and Heymann *et al.* (2005 ▸). Alternatively, they are defined by the rotation operators (Frank *et al.*, 1981 ▸), quaternions (Heymann, 2001 ▸) or Rodrigues coordinates (Punjani *et al.*, 2017 ▸). For the goals mentioned above, it is more convenient to characterize them by the straight line orthogonal to each projection, *i.e.* its direction or view. Each view can be identified by the respective values of the spherical angles or by the point of its intersection with the unit sphere, *i.e.* by the respective unit vector. A uniform distribution of such points on the spherical surface, with no missed regions, means that the respective Fourier coefficients, especially the higher-resolution ones, are determined everywhere in the Fourier space and with a similar accuracy, considering possible measurement errors, errors in the estimates of the projection direction *etc*. Information about the Fourier coefficients (obviously, except their indices) is independent of the rotation about the projection line. Also, two opposite views correspond to two mirror projections and contain the same information.

The frequency of views can be expressed numerically in several different ways:

(i) Calculate how much each particular view is separated from other views.

(ii) Calculate how many different views are close to each other and how close.

(iii) Calculate how often a given or similar view is represented in the list of views.

A procedure following the first way has been suggested by Orlov *et al.* (2006 ▸). An example of the second approach using distance graphs for the points on a sphere is described by Fan & Zhao (2019 ▸). The third approach can be addressed by calculating histograms on a sphere using bins of an equal or near-equal surface area, *e.g.* as reported by Scheres (2012 ▸).

Both numeric and visual expressions of the frequency of views help to understand the eventual existence of dominating views, also known as preferred orientations. Different types of visualization can be suggested. *BKPR* (Orlov *et al.*, 2006 ▸) re­presents individual views by points in a geographic Mollweide projection of a sphere. A combination of the software *Relion* (Scheres, 2012 ▸) with *Chimera* (Pettersen *et al.*, 2004 ▸) uses bars on the surface of a sphere, with the bar length pro­portional to the view frequency. *cryoSPARC* (Punjani *et al.*, 2017 ▸) represents a distribution by a colour map with two Euler angles as the Cartesian coordinates, and *cisTEM* (Grant *et al.*, 2018 ▸) uses the equidistant (by spherical angles) projection of the top hemisphere.

The methods and algorithms of both frequency calculation and its mapping as realized in the different programs are doc­umented rather poorly, probably being considered as auxiliary and technical. Another common weak point is that, when printing maps of distributions calculated as described above or drawing a non-interactive 2D projection of the pre­sentation as bars on the spherical surface, these images represent information rather qualitatively, distorting the relative area of the spherical surface regions.

Addressing these problems, we identified several appropriate computational tools largely used in other research fields. The first tool we are interested in is a projection of a hemisphere to a plane conserving, or approximately conserving with a high accuracy, the surface area. Several such projections are known, for example, the Lambert azimuthal equal-area projection, the dymaxion mapping (‘dynamic maximum tension’) and the Snyder equal-area projection (Snyder, 1992 ▸). The first of these was proposed as early as 1772, and its comprehensive presentation including proofs can be found, for example, in the work of Roşca (2010 ▸). The dymaxion mapping, also known as Fuller’s projection, was developed in the 1940s and 1950s, and some of its formal considerations can be found in articles by Gray (1995 ▸) and Crider (2008 ▸). More projections are described by Snyder (1993 ▸) and by Grafarend & Krumm (2006 ▸).

Our second computing tool is the construction of a regular grid on a spherical surface, also known as sphere pixellation. Given a projection conserving surface area, this geometric problem becomes equivalent to the construction of a regular grid on a plane. Several such algorithms are known, for example those by Roşca (2010 ▸), Cox & Flikkema (2010 ▸) and Masset (Masset *et al.*, 2011 ▸; Jacques *et al.*, 2015 ▸), and in particular *HEALPix* by Górski *et al.* (2005 ▸) used previously in *Relion* (Scheres, 2012 ▸); see also references therein.

Selecting appropriate algorithms among these multiple options, we have developed the program *VUE* to calculate the frequencies of views (of 2D projections), to quantify and visualize their distributions by a plane image, and eventually to produce a new set of views distributed more uniformly. Working on this project, we addressed several goals:

(i) Develop a *stand-alone* tool which can provide various types of information about the distribution of projections, in both numeric and graphical forms.

(ii) Make the graphical representation *quantitatively correct*, *i.e.* such that the area of the 2D regions in the plane image is proportional to the area that they cover on the spherical surface.

(iii) *Describe explicitly and in detail* the methods and algorithms used for the computations, making them reproducible if required.

(iv) Propose multiple choices of options for different steps of the procedure, allowing the user to produce *images adapted to their personal taste* in terms of details, colours *etc.* and to analyse *the role of different parameters*.

(v) Make this program composed of small modules, allowing one to routinely add other options if required.

(vi) Prepare a tool to analyse, in a separate project, the role of weighting schemes optimizing the 3D reconstruction.

In particular, this program may be considered not only as a research and illustrative tool but also as a methodological and training one.

## 2. Methods and algorithms

### 2.1. Geometric tools

#### 2.1.1. Coordinate systems, rotations and views

**Figure 1:**
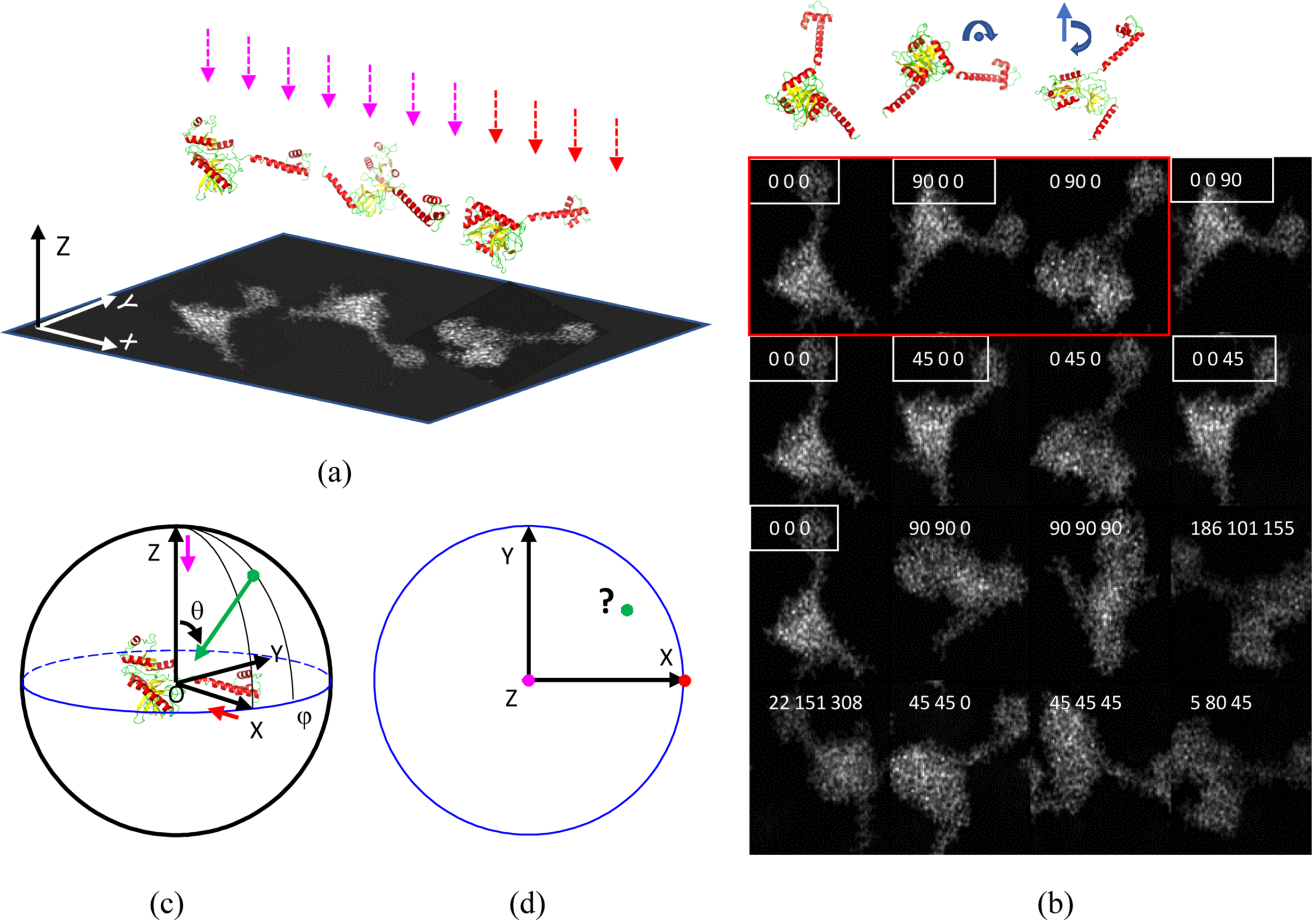
Schematic projections of an IF2 molecule. (*a*) The molecule in three different orientations projected along the **OZ** direction chosen as opposite to the beam direction. (*b*) Several projections of the IF2 molecule. The numbers on each projection are its respective Euler angles in the standard convention. Seven projections, marked by white frames and corresponding to different Euler angles, are actually the same or equivalent. This is an example of an over-represented view. The three projections inside the red frame correspond to the projections in panel (*a*); the respective models are shown above. (*c*) The reference model corresponding to Euler angles (0, 0, 0) is at the origin. The magenta arrow indicates the view along **OZ**, as in panel (*a*). The red arrow indicates the view along **OX** and corresponding to the molecule rotated by (0, 90, 0) in panels (*a*) and (*b*). The green arrow corresponds to a view along some arbitrary direction with the spherical angles θ (with **OZ**) and φ (azimuthal angle). (*d*) A plane representation of the views from panel (*c*) with the position of the green point to be defined. The coordinates of the coloured points, representing the views, depend on the method of representation. The program *PyMOL* (Schrödinger LLC & DeLano, 2020 ▸) was used to produce the IF2 models.

The description of the projections, rotations and orientations is one of the confusing points in cryo-EM due to the different choices of parameters used by different programs. Frequently, these choices are documented incompletely. Let us suppose that the sample contains a single type of molecule or complex, all in exactly the same conformation (the first prior mentioned in the *Introduction* [Sec sec1]). Each original cryo-EM image contains a large number of orthogonal projections of the object oriented differently with respect to the beamline and to the plane orthogonal to it. Figs. 1 ▸(*a*) and 1 ▸(*b*) give schematic illustrations, with a highly asymmetric model, of the structure of eubacterial translation initiation factor 2 (IF2) (Simonetti *et al.*, 2013 ▸). The plane of projection and the beam direction define the global fixed basis 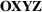 of the Cartesian coordinate system with the axis 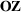 against the beam direction. A molecule in one of its orientations is chosen as a reference for which the 3D map of the scattering electrostatic potential is calculated. All other orientations are defined by the rotation operator applied to this molecule, keeping the basis 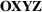 . While such an operator is defined unambiguously by the matrix 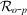 in 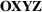 according to the standard mathematical rules, independent parameters describing such a rotation can be chosen differently. The cryo-EM convention (Heymann *et al.*, 2005 ▸) describes a rotation by the Euler angles in the mobile basis associated with the reference molecule and coinciding initially with 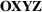 . It is known that several different combinations of Euler angles may result in the same projection [Fig. 1 ▸(*b*)]; this is one reason why some software programs use alternative rotation descriptions, as mentioned in the *Introduction* [Sec sec1]. Possible rotation descriptions and their interrelations are discussed by Urzhumtseva & Urzhumtsev (2019 ▸).

For the 3D reconstruction, it is convenient to consider each projection of the rotated molecule along 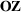 differently, as a projection of the initial fixed molecule but along the respectively modified direction or view. For example, a projection along 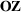 of the molecule described by Euler angles (0, 90, 0) according to the cryo-EM convention (which results in the clockwise rotation of the molecule by 90° about axis 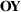 , from 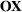 to 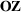 , a direction opposite to the trigonometric one) is equivalent to the view of the non-rotated molecule along 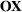 [Fig. 1 ▸(*c*)]. Each direction is characterized by a vector, initially coinciding with 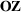 and being rotated by the inverse rotation operator. The unit vector along this direction, the view, is the third column of the inverse matrix 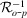 , whatever the rotation parameterization is. For this reason, the procedure described below first of all converts each set of 2D projection parameters into the respective rotation matrix and extracts the unit view vector.

The task is to produce, in a plane, a representative spatial distribution of such view vectors, for example to show the ensemble of the points of intersection of such vectors with a unit sphere [points in Fig. 1 ▸(*d*)].

#### 2.1.2. Lambert projection

There exist a large number of mappings from a sphere to a plane possessing different geometric properties. Our principal requirement is conservation of the surface area. Among several such methods, that of our choice should be simple by itself and give the simplest possible projection, the easiest to manipulate. For this reason, we selected the Lambert azimuthal equal-area projection, also known as the Lambert zenithal equal-area projection, which we refer to herein as the Lambert projection and which we provide a reminder of below. Since the projection following the vector 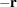 gives just the mirror view with no extra information compared with the projection following 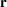 , we deal with a projection of a hemisphere (we arbitrarily choose the upper one, 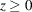 ) selecting which one of 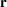 or 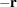 belongs to it.

**Figure 2:**
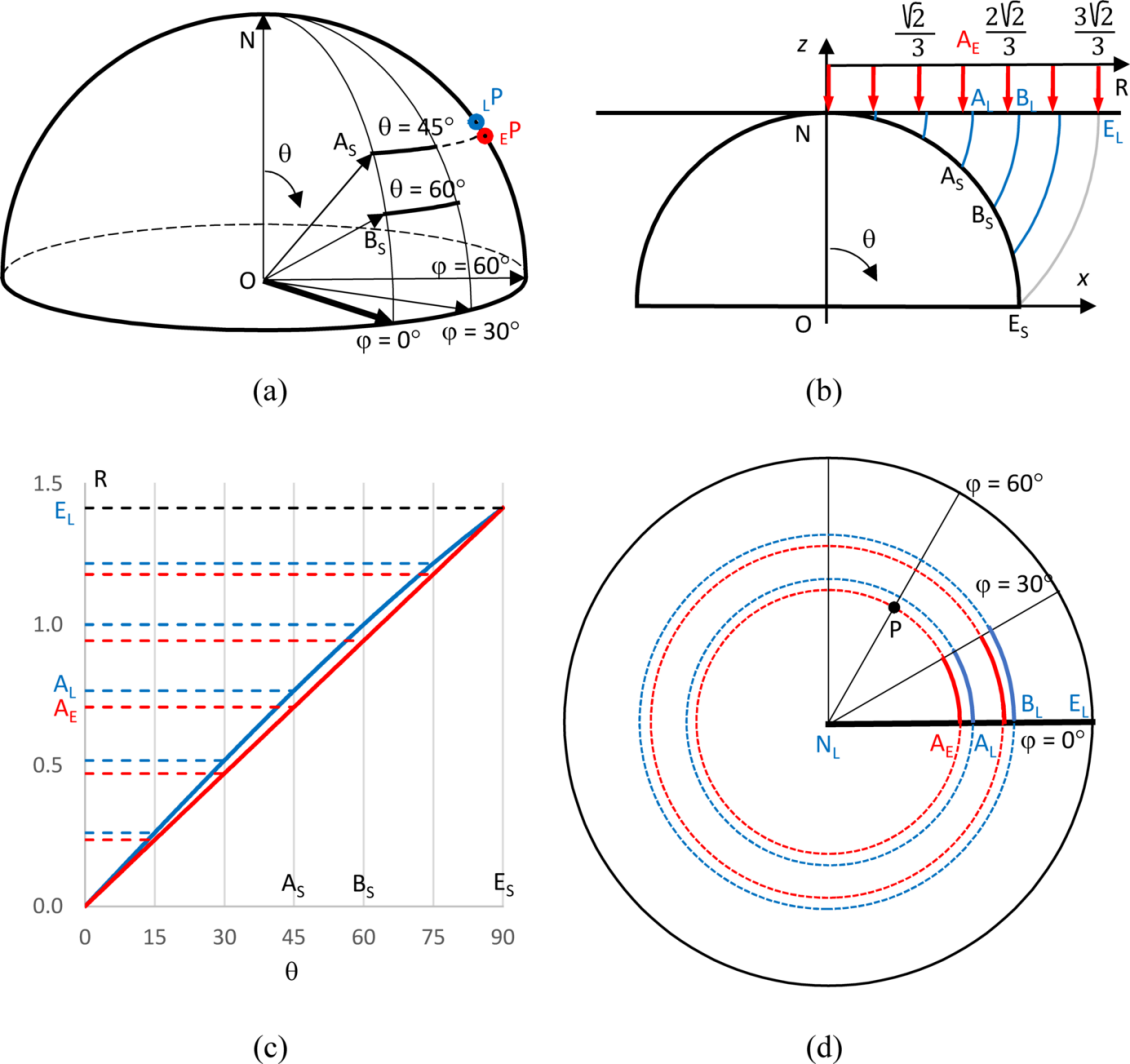
An illustration of the Lambert projection of the top unit hemisphere. The indices S, L and E indicate, respectively, points on the sphere, in the Lambert projection and in the ‘standard’ angular-equidistant projection for which the distance of the point from the centre is proportional to the spherical angle. (*a*) A three-dimensional illustration of some points on the unit sphere shown, in projection, in the other images. Points _E_P and _L_P illustrate the back projections onto the hemisphere of the point P in the plane with *R_n_* = 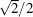 and φ = π/3 shown in panel (*d*). (*b*) The Lambert projection conserves the distance from the north pole N. Red arrows indicate, for comparison, the angular-equidistant projection, with *R_n_* = 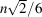 , of the points with θ = *n*π/12, *n* = 0, 1,…, 6. (*c*) The distance *R* from the centre for the angular-equidistant projection, in red, and for the Lambert projection, in blue, as a function of θ, quantifying the information in panel (*b*). (*d*) The angular-equidistant projection, in red, and the Lambert projection, in blue, of the arcs shown in panel (*a*).

Let a point A_S_ = (*x*, *y*, *z*) be defined by its Cartesian coordinates on the top unit hemisphere, 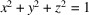 , 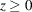 , and let 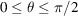 and 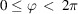 be the respective spherical angles [Fig. 2 ▸(*a*)]. These angles should not be confused with the Euler angles if the latter are notated similarly. The Lambert projection of a point A_S_ onto the plane 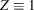 is the point A_L_ = (*X*, *Y*) with the same azimuthal angle 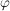 and with the same distance from the north pole N: |**NA**_L_| = |**NA**_S_| [Fig. 2 ▸(*b*)]. The coordinates of A_S_ are

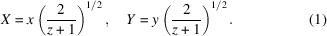

Thus, the hemisphere is projected onto a disc, the north pole is projected onto the centre of this disc and the points E_S_ on the sphere’s equator are projected onto an outer circle of radius 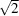 : |**NE**_L_| = |**NE**_S_| = 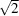 [Fig. 2 ▸(*b*)]. Arcs 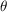 = const on the sphere are projected onto arcs of concentric circles of radius 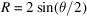 , while arcs 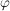 = const on the sphere are projected onto straight radial lines [Figs. 2 ▸(*c*) and 2 ▸(*d*)]. For the Lambert projection, the circles corresponding to 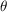 taken with a given step are compressed to the periphery compared with the circles obtained by projections with a fixed step by the spherical angle θ (the ‘angular-equidistant’ projections), for which the radius is 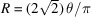 [Fig. 2 ▸(*c*)]. Here, the coefficient in parentheses is chosen to conserve the total spherical surface area of the hemisphere. In particular, the angular-equidistant projection shrinks by 1.18 times the area inside the circle for 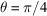 compared with the area of the respective circle in the Lambert projection; this value is 1.13 for a circle with 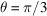 . Using a relatively fine grid for the frequency analysis, *e.g.* that with a default value of *M* = 90 (see Section 2.2 below), shifts the position of the bin for a view with 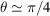 by five to six grid cells between these two types of projection.

The inverse projection, that of the disc onto the unit top hemisphere, converts a point with coordinates (*X*, *Y*), such that 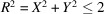 , to a point with coordinates

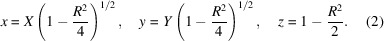




While the direct Lambert projection is important to plot the calculated frequency map, the inverse projection is crucial to transfer a uniform grid inside a disc (Section 2.2) onto a grid on the unit sphere.

### 2.2. Uniform grids

A uniform grid on a sphere is a grid such that all its cells have equal, or near equal, surface area. Because the Lambert projection conserves the surface area, it is possible to build first a uniform grid on a disc, which is a simpler task, and then to project it onto the sphere. The current version of the program can use two uniform grids described below. Both of them are constructed starting from concentric circles on a disc and are easy to manipulate.

**Figure 3:**
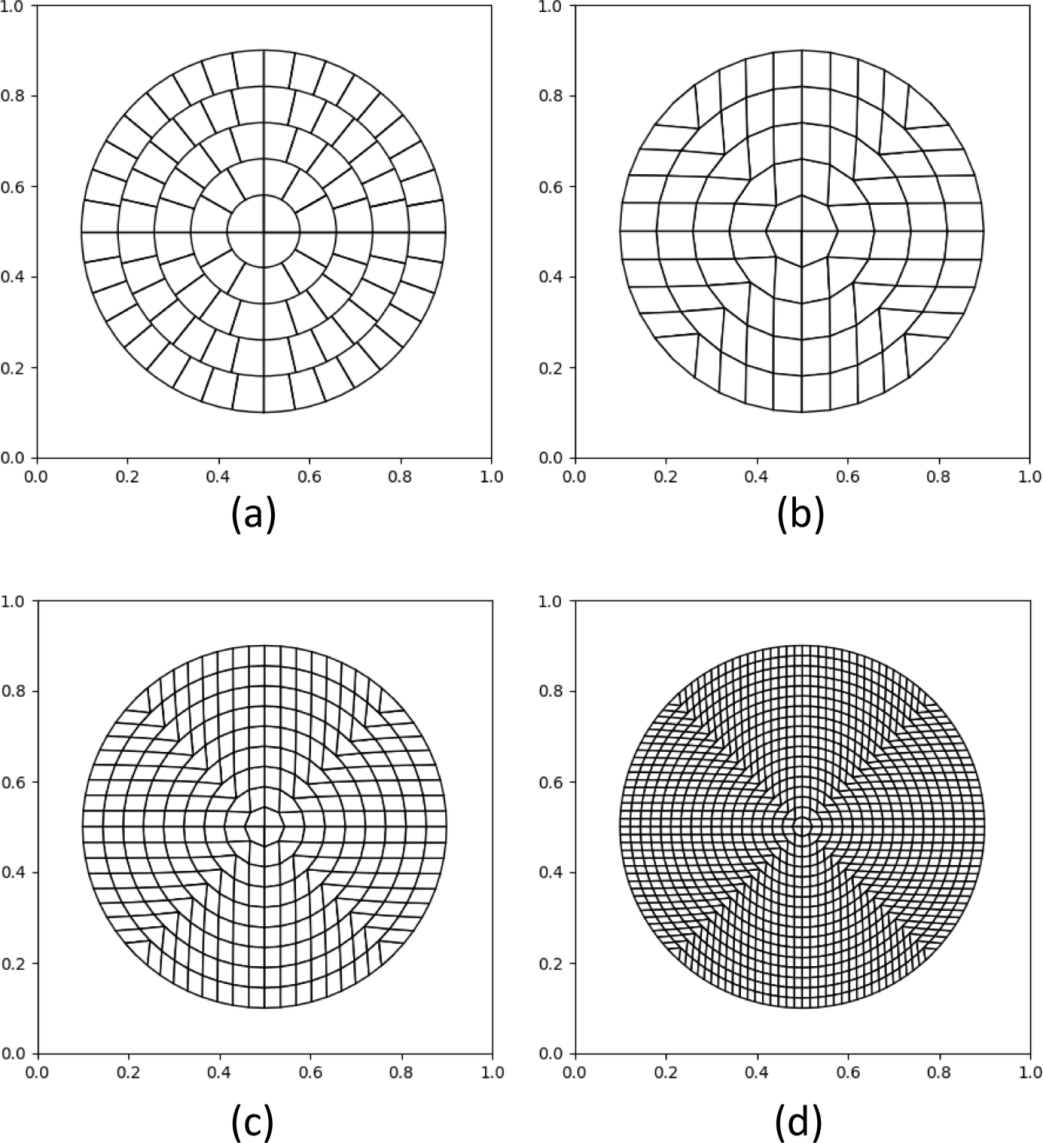
Examples of uniform grids on a disc. Grids according to (*a*) Masset *et al.* (2011 ▸) with *N* = 4 and (*b*)–(*d*) Roşca (2010 ▸). *M* is equal to 5 in panels (*a*) and (*b*), 9 in panel (*c*) and 18 in panel (*d*).

Masset *et al.* (2011 ▸) constructed *M* concentric equidistanced circles inside a disc of radius 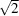 . The first layer, 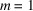 , *i.e.* the internal disc, is divided into *N* equal sectors where *N* is a parameter. Each subsequent circular layer *m* is divided into 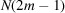 equal parts following the polar angle 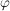 [Fig. 3 ▸(*a*)].

Roşca (2010 ▸) also started from constructing *M* concentric equidistanced circles inside a disc of radius 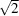 . Each circle 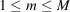 is divided into 8*m* equal arcs with their bounds indexed by 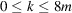 . For polar angles φ < 45°, *i.e.* for 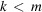 , the near-straight lines *k* = const form two opposite sides of a grid cell, the two other sides of which are formed by the respective circles [Fig. 3 ▸(*b*)]. The grid in other sectors, φ > 45°, is built by mirror symmetry about the coordinate axes and the diagonals; the diagonals themselves are not cell borders. Appendix *A* [App appa] describes this in more detail, mentioning also the computational advantages and disadvantages of these grids.

For both types of grid, it is convenient to identify each cell by the largest values of the integer indices (*m*; *k*) over all four vertices (Appendix *A* [App appa]). The cells on the spherical surface have the same indices as their disc projections. For further calculations, we denote by 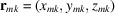 the centre of the cell *mk* on the sphere, that with the indices (*m*; *k*). We also denote by 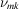 the frequency of the projections corresponding to this cell, the values of which are to be calculated as described below.

### 2.3. Calculation of the views’ frequencies

#### 2.3.1. Point representation of a view

The input cryo-EM data used for the 3D reconstruction, in addition to the images themselves, include rotation parameters which explain how the object should be rotated in order to correspond to a given projection or, inversely, how a given projection should be rotated to correspond to the respective object image. For example, a file in the star format produced by the program *Relion* (Scheres, 2012 ▸) describes this by three parameters *phi*, *psi* and *tilt* which correspond to the Euler angles according to the convention defined by Heymann *et al.* (2005 ▸, 2006 ▸).

Whatever the parameterization is, the program first converts the rotation parameters into the projection direction, the view. Each view *n* is characterized by the coordinates 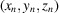 of the respective point 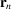 on the unit sphere as described above. If the point 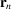 belongs to the bottom hemisphere, 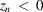 , the point for the equivalent mirror view 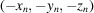 belongs to the top hemisphere and is used instead of 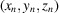 .

#### 2.3.2. Symmetries

For symmetric objects, a statistical analysis may be distorted in two opposing ways. On one hand, the presence of views covering only a part of the sphere may allow a full representation of all views and recovery of all Fourier coefficients equally well. However, a statistical analysis of the formally present views will indicate a highly uneven distribution with large empty regions. On the other hand, the available views may result in a non-uniform covering of the sphere if considering all their symmetry-related copies.

For this reason, before starting the statistical analysis of the views, the program may expand their set over symmetries of the selected spatial point group; the default group is *C*_1_. The directions of the two principal axes of the point group, *i.e.* that aligned with the principal rotation axis ( 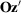 ) and that in the orthogonal plane ( 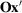 ), do not necessarily coincide with those of the Lambert projection and can be modified by the user.

#### 2.3.3. Histogram calculation

Given a uniform spherical grid, one can calculate the frequency 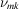 of views similarly to the calculation of the usual one-dimensional histograms. An optimal choice of the grid size [see *e.g.* Sturges (1926 ▸) and Freedman & Diaconis (1981 ▸)] is important since an overly coarse grid does not catch the details of the distribution, merging into a single bin close but different events. Inversely, an overly fine grid may make the calculated distribution too fluctuating and with a statistically unconfident bin content, especially when the number of contributions is of the same order as the number of bins.

To assure representative, accurate and robust results, the program *VUE* uses the approach referred to as the kernel density estimation. A kernel is a smooth non-negative valued function which decreases monotonically from the origin, extends over several bins and has a unit integral value. A normalized Gaussian function is an example. Schematically, when calculating a conventional integer-valued histogram, a unit value is added to the bin to which the given event belongs. Instead, when working with a kernel, it is first centred at the point where the event has occurred. A real value equal to the integral of this kernel over each bin is then calculated and added to this bin’s counter. With such a mode of calculation, the resulting real-valued histogram is more robust with respect to the grid size, to the number of contributions and to inexactitude in the event’s position (orientation of the 2D projections). Previously, such an approach has been used efficiently in macromolecular crystallography (Lunin, 1988 ▸; Afonine *et al.*, 2015 ▸).

Following this approach and ignoring bins with negligibly small integral values, every view 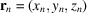 adds its contribution to a few cells *mk* of the uniform grid. This contribution is calculated according to the value of the distance *d* from the point 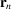 to the centre 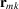 of the respective 2D bin on the sphere within the given distance limit, 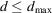 . Since the view contribution is local, the distance limit 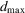 is taken to be small.

For two points 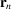 and 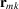 on the surface of a unit sphere and close to each other, the distance *d* between them is close to the spherical distance, *i.e.* to the length of the great circle joining these points. In turn, this length is numerically equal to the angle 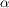 , in radians, between the respective unit vectors 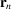 and 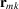 , making 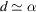 . For small angle values, 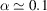 or smaller, this gives the relation

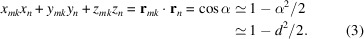




Overall, with (3), the contribution 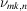 of a view *n* to the cell *mk* is calculated as the 2D Gaussian function of the distance *d* using the Cartesian coordinates of the points 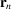 and 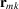 at the unit sphere,

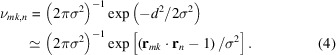




The parameter 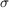 is taken such that at the limit distance 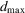 the function value is 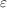 times smaller than that at the centre of the Gaussian; 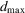 and 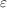 are parameters of the procedure. Using the ‘blurred’ contribution (4) implicitly reflects uncertainties in the values of the projection’s direction.

Actually, two views at a distance *d*, *i.e.* mutually disoriented by a respective small angle 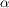 , may contribute to different higher-resolution Fourier coefficients but to the same low-resolution ones. In that sense, parameter 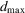 is more than a computational ‘handle’. Calculating the views’ frequency with larger values of 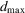 illustrates over- and under-representation influencing low-resolution Fourier coefficients, those defining the shape of the reconstructed object. Inversely, using smaller *d*_max_ values shows a misrepresentation of the higher-resolution coefficients responsible for specific details and their accuracy and correctness. In our tests, with the grid number *M* = 90, we varied 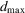 in the limits 1–3°.

In order to compare the total contribution

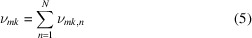

of all views to the cells *mk* with that for the uniform distribution, the values 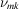 are normalized, being multiplied by the single-cell area which depends on the type of the chosen uniform grid and its parameters (Appendix *A* [App appa]), as all the views are uniformly distributed over the surface.

#### 2.3.4. Frequencies of individual views

Once the 2D histogram of the views’ distribution on the spherical surface has been calculated, the frequencies of the individual views can be defined from it by various approaches. For a view *n*, the simplest way to assign its frequency 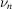 is taking it equal to that of the grid cell to which this view belongs, *i.e.* 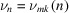 . Obviously, different kinds of interpolation from neighbouring cells are possible.

After calculating the frequencies 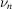 of all *N* individual views, these values are scaled by 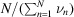 , making their sum equal to *N*. With such a scale, for a uniform distribution each view would have a frequency equal to one. The calculated normalized frequencies 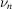 can be illustrated by a diagram, saved in an output file and/or used for different goals, *e.g.* to down-weight or filter preferred orientations.

### 2.4. Weighting of views

To reduce artefacts caused by a non-uniform distribution of views, their normalized frequencies 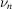 can be used to filter or weight misrepresented views during 3D image reconstruction [see *e.g.* Scheres (2012 ▸), Sorzano *et al.* (2021 ▸), and references therein]. The simplest weights are the values inverse to the normalized frequencies. The *VUE* program uses a more general weighting scheme with the parameters 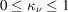 and 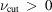 ,

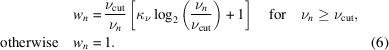

This includes the previous scheme as a particular case when 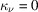 and 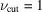 . The default parameter values can be modified by the user; 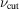 should be above the minimum 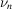 value, but it is automatically corrected otherwise. Scheme (6) is based on the observation that the amount of information increases logarithmically with the amount of cryo-EM data (Stagg *et al.*, 2014 ▸), similar to what occurs in X-ray crystallography (Urzhumtsev *et al.*, 2009 ▸). For this goal, the factor in square brackets in (6) increases the weight for highly represented views, reflecting also more confident orientation parameters.

Other weighting schemes can be implemented routinely.

### 2.5. Updating of views

As an example of using the calculated frequencies, the program can reduce the number of over-represented views, those above a defined threshold level. This is done by selecting and randomly removing the views according to the weights calculated above. A more advanced option allows the program, after removing some over-represented views, to complete the under-represented views with the goal of approximately conserving the total number of projections in the input file. For each under-represented view, extra records are generated referring to the same experimental 2D projection, with their number calculated according to the previously calculated weight. The orientation parameters of each such artificially generated ‘daughter view’ differ from those of the ‘mother view’ by some small perturbation factor defined by the user. Such an artificially extended data set explicitly models uncertainties in the view’s orientation under the hypothesis that the true signal will be conserved while the random noise in slightly different views will cancel each other after addition of their contributions. This modelling is in some ways similar to a statistical weighting of crystallographic maps [see *e.g.* Blow & Crick (1959 ▸) and Read (1986 ▸)]. Eventually, this may slightly blur the map details while making the new maps less noisy and more readily interpretable. These maps are complementary to the maps calculated directly with the initial non-uniform set of views and can be used together with the latter.

The choice of correction mode and of its parameters depends on the 3D reconstruction method. Comparison of the weighting and view processing schemes based on frequency, weights and eventually on other types of information will be discussed elsewhere.

## 3. Results

### 3.1. Data used

Uniform grids make it routine to extract different types of information, to calculate various kinds of statistics and to express it in different forms. This includes both images and a numeric output, as discussed below. To illustrate the options of the program, several experimental data sets measured for the human ribosome have been used.

The first two data sets, called Set1 and Set2, were obtained using the latest-generation Titan Krios G4 (TFS) 300 kV electron microscope, as described previously (Fréchin *et al.*, 2023 ▸). These data sets were composed of about 74 000 and 139 000 2D projections, respectively. The third data set, called Set3 and composed of about 337 000 2D projections, was measured using the in-house Titan Krios G4 cryo electron microscope with a Falcon 4i camera (300 kV acceleration voltage).

**Figure 4:**
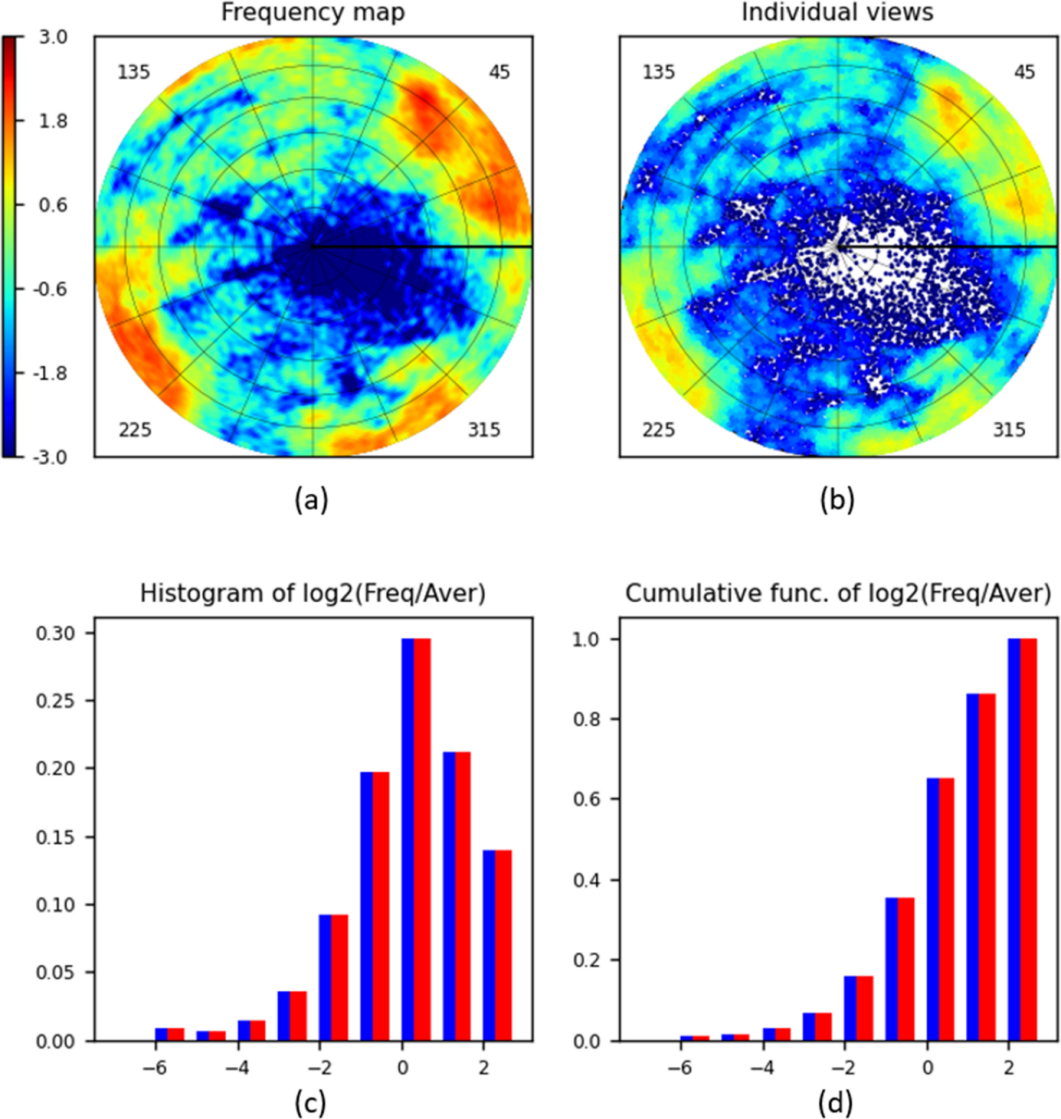
*VUE* diagrams calculated for the human ribosome data set Set1 (∼74 000 projections). The default *jet* colour scheme from *MatPlotLib* (Hunter, 2007 ▸) is used both for the maps and for individual views. The colour bar and the histogram argument are given on the log_2_(ν_*mk*_/ν_uniform_) scale with the zero value corresponding to the uniform distribution. (*a*) The distribution map calculated with *d*_max_ = 3°. (*b*) Individual views. (*c*) A histogram of the cell frequencies and (*d*) the respective cumulative diagram, showing the frequency distributions for all cells (blue) and for non-empty cells (red).

Fig. 4 ▸ shows typical diagrams prepared by the program *VUE* and Sections 3.2–3.7 [Sec sec3.4] [Sec sec3.5] [Sec sec3.6] [Sec sec3.7] comment in detail on their content and interpretation. The subsequent figures and sections give more illustrations of different situations, options and parameter values.

### 3.2. Frequency map

The map of view frequency represented by its Lambert projection is the principal diagram produced by the program. Fig. 4 ▸(*a*) shows such a map calculated for Set1. The size of each region is proportional to the respective surface area on the sphere. The cells of the uniform grid are shown in different colours according to the associated grid frequency value. The colour scale is a logarithmic one, similar, for example, to that used in *cryoSPARC* plots (Punjani *et al.*, 2017 ▸). The actual scale is 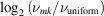 , comparing the frequencies with the average normalized value; increasing the scale value by one corresponds to a two-times higher frequency. As expected from the histogram for Set1 [Fig. 4 ▸(*c*)], there are low-frequency views, and the map in Fig. 4 ▸(*a*) shows that they are merged into a single large region around the centre of the diagram.

By default, the axis of the Lambert projection coincides with the beam direction. This can be reassigned by the user, and the views will be recalculated and shown for the respective hemisphere following the new chosen direction.

Fig. 4 ▸ illustrates the results obtained with the grid calculated according to Roşca’s approach with *M* = 90 and with *d*_max_ = 3°. As expected for such a relatively large *M*, the diagrams obtained using the grid proposed by Masset *et al.* (2011 ▸) were indistinguishable from the previous ones. Extra bilinear interpolation applied when using the Roşca grid also made no difference (results not shown).

### 3.3. Distribution of individual views

The distribution of individual views is represented by dots in the Lambert projection of their respective positions 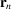 on the unit hemisphere. For Set1 [Fig. 4 ▸(*b*)], relatively isolated dots are close to the centre of the diagram, *i.e.* corresponding to the poorly represented directions nearly perpendicular to the plane of the image. The (optional) superimposed reference grid shows that, more precisely, this is the region about the view characterized by the spherical angles θ ≃ 15° and φ ≃ 340°.

A disadvantage of this type of plot is that the dots for close views may superimpose, and slightly over-represented views can be barely distinguished from severely over-represented ones. A possible solution is to apply, by the user’s choice, different colours for dots corresponding to views with different frequencies, as is done in Fig. 4 ▸(*b*). In general, this plot is appropriate for relatively small sets of views, as illustrated below.

### 3.4. Histograms

Conventional histograms of the values calculated for spherical grid cells [Fig. 4 ▸(*c*)] and their cumulative function [Fig. 4 ▸(*d*)] reflect the overall information about the distribution of views and indicate the eventual presence of dominating or missed views, ignoring their spatial position. This is a kind of fast preliminary step, giving quantitative information that is easy to represent and to analyse before looking at a detailed frequency map. We chose the bounds of the histogram bins on a logarithmic scale, agreeing also with the observations by Stagg *et al.* (2014 ▸). In the program *VUE*, the bins’ bounds are taken as 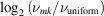 , *i.e.* they each double with respect to the previous one, with zero corresponding to the uniform distribution.

The histogram calculated for Set1 [Fig. 4 ▸(*c*)] shows that about 0.3 of the total number of cells have a frequency close to 2^0^ = 1 (the highest bar in blue). Approximately 0.2 of the cells have a frequency of about 2^1^ = 2, and roughly the same number have a frequency of about 2^−1^ = 0.5 (the bars in blue next on the right and the left). The histogram also tells us that there are no cells with a high relative frequency, above 2^2^ = 4 (*i.e.* there are no strongly dominant views; about 0.14 of the total number of cells have frequencies from 2 to 4, as the right-most bar shows). There are very few cells with frequencies of the order of 2^−6^ ≃ 0.015 or lower (left-most bar) compared with the mean value. The exact number of views in different bins can be found in the file VUE.log.

In unfavourable situations (*e.g.* Section 3.6) some grid cells may stay empty, with their frequency equal to zero. For this reason, the program calculates and plots the histograms both taking such cells into account (histogram in blue) and excluding them (histogram in red). Figs. 4 ▸(*c*) and 4 ▸(*d*) show that for Set1 these histograms fully coincide with each other; this means that there are no totally empty grid cells.

### 3.5. Ribosome, extended data set

**Figure 5:**
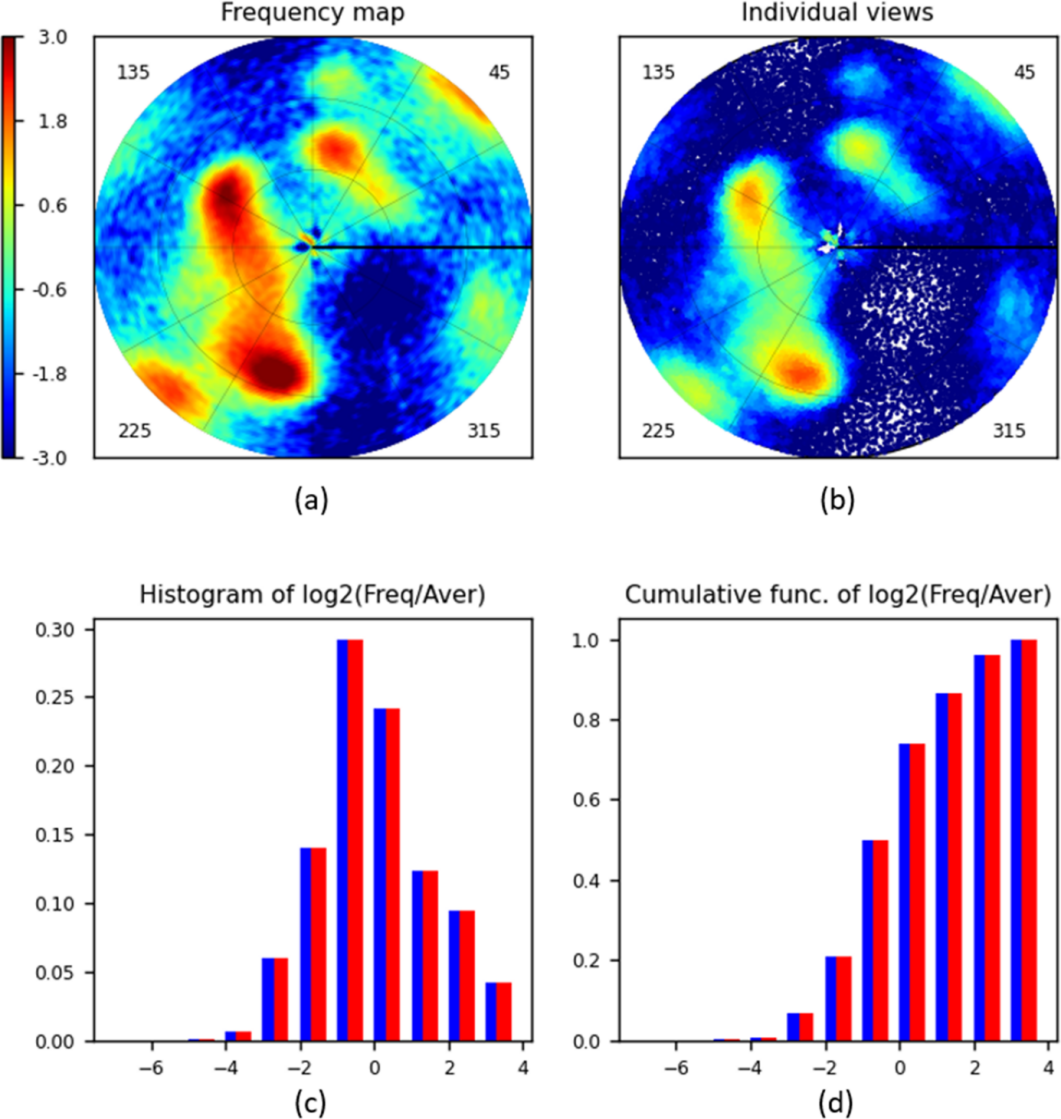
*VUE* diagrams calculated for the human ribosome data set Set2 (∼139 000 projections). The types of diagram and their parameters are the same as in Fig. 4.

Fig. 5 ▸ illustrates the results of the program applied to a larger data set, Set2, completed up to about 139 000 projections with the goal of filling the previously missed views. Indeed, the previously empty region is covered quite well. However, the diagrams show that most of the added views correspond to two directions characterized by, respectively, the spherical angles (θ ≃ 40°, φ ≃ 150°) and (θ ≃ 50°, φ ≃ 250°). While the regions occupied by these views are relatively small, the view frequency in these regions is about 2^3^ = 8 larger than the mean value. The presence of such dominant views probably makes the respective 3D reconstruction suboptimal. Fig. 5 ▸(*c*) also shows that the addition of these over-represented views reduces the frequency for most of the cells from 1.0 to 0.5 compared with the mean value (the highest peak of the histogram corresponds to 2^−1^ = 0.5). Most important is that, for this set, nearly all frequencies are above 2^−3^ ≃ 0.1 [Fig. 5 ▸(*c*)], showing that the goal of completing the data has been achieved.

### 3.6. Ribosome, reduced set

**Figure 6:**
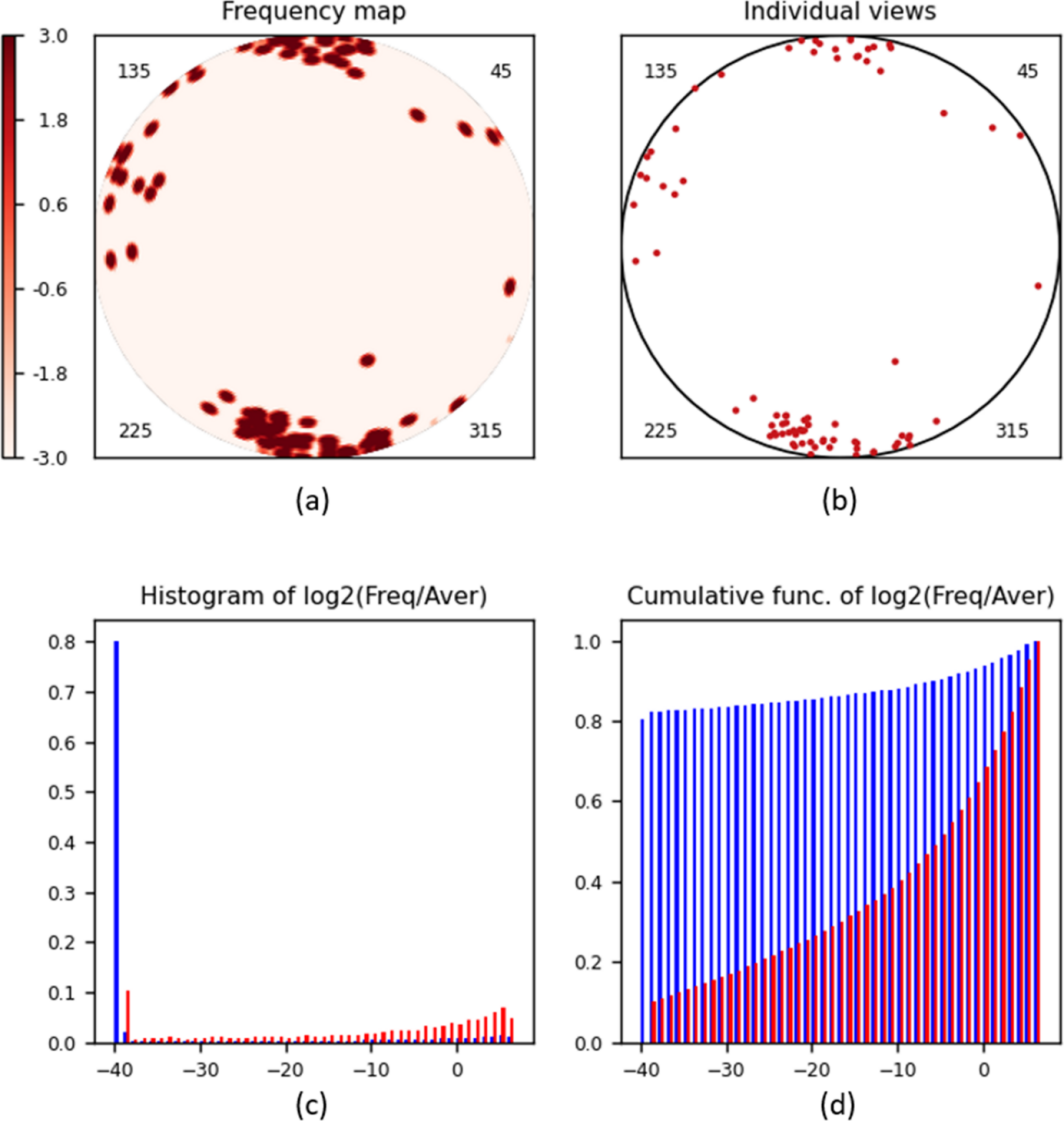
*VUE* diagrams calculated for a small subset of Set2 (80 projections). The map is shown with the *Reds* colour scheme, and individual views are shown with a unique colour. The histograms are calculated within large bounds. Other parameters are the same as in Fig. 4.

Fig. 6 ▸ shows the diagrams for a very small subset of Set2 composed of only 80 projections calculated with the same value *d*_max_ = 3°. Using the same colour scheme as above produces a map mostly in dark blue (not shown). Instead, for small sets it is more practical to use a *MatPlotLib* colour scheme like *Reds*, with the intensity increasing with the frequency value [Fig. 6 ▸(*a*)]. This time, since the views are mostly separated in space [Fig. 6 ▸(*b*)], they may be displayed in the same colour. For the same reason, the frequency map [Fig. 6 ▸(*a*)] looks like the views themselves [Fig. 6 ▸(*b*)], just with each ‘individual view’ being blurred around its position. A few peaks are merged with each other, giving for some cells a frequency of about 2^6^ = 32 with respect to the average one [the right-most bars in Fig. 6 ▸(*c*)].

A high peak at the left of the histogram calculated for all cells [blue bars in Fig. 6 ▸(*c*)] indicates that about 80% of the spherical surface is not covered at all. For this reason, the histogram calculated for non-empty cells only (red bars) is different from that in blue. The histogram, being calculated on purpose within very large bounds, shows that about 10% of non-empty cells (left-most red bar) have near-zero frequencies; they are cells at the periphery of isolated views, *i.e.* of practically every view. Except for these two highest bars, the bars are hardly distinguishable in the histogram [Fig. 6 ▸(*c*)] and the cumulative histogram [Fig. 6 ▸(*d*)] represents this information more clearly.

### 3.7. Ribosome, data set Set3

**Figure 7:**
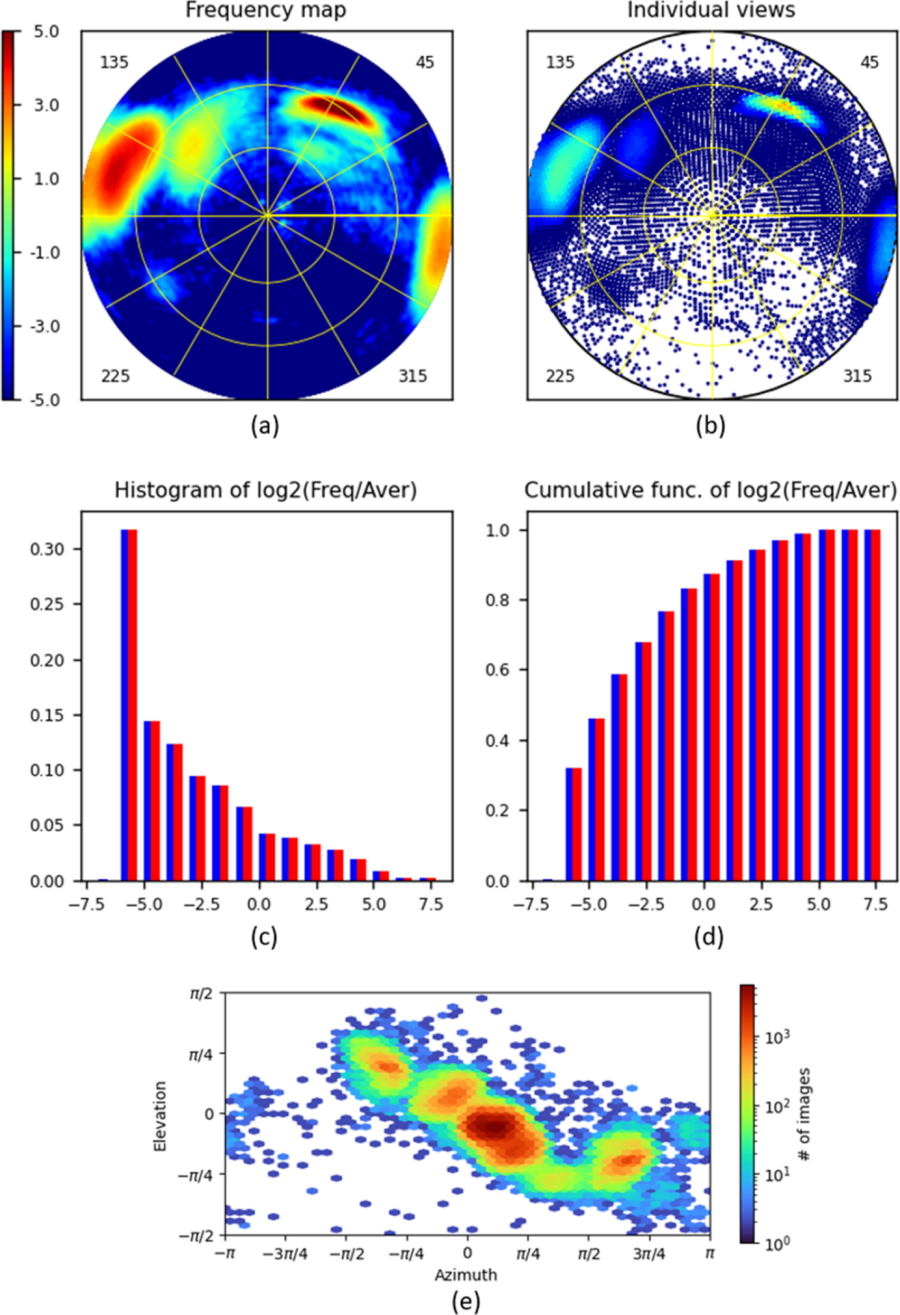
*VUE* diagrams calculated for data set Set3 (about 337 000 projections). The default *jet* colour scheme from *MatPlotLib* is used. The types of diagram are the same as in Fig. 4, although the scale bar limits for the frequencies in panels (*a*) and (*b*) are slightly different. (*e*) A representation of the distribution of views prepared with *cryoSPARC*, for comparison with panel (*a*).

Fig. 7 ▸ illustrates an application of the program to quite a large data set, Set3, composed of more than 337 000 projections. A straightforward 3D reconstruction with Set3 resulted in a deformed image (not shown; project in progress). This can be explained, at least partially, by the fact that Set3 contains four regions of extremely dominant views, and moreover, two of them are close to each other at opposite ends of a diameter of the projection [Fig. 7 ▸(*a*)]. It also shows that there are missed views in the direction roughly orthogonal to that for the preferred orientations. On the other hand, although Fig. 7 ▸(*e*) prepared by *cryoSPARC* may make one believe that the views with approximately 70° ≤ θ ≤ 90° are represented much less frequently than the other views (see the horizontal layer close to 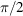 ), this is not the case.

The histograms [Figs. 7 ▸(*c*) and 7 ▸(*d*)] indicate a huge difference in frequency of views corresponding to different regions of the spherical surface, of the order of 2^13^ ≃ 10^4^. The distribution calculated with *d*_max_ = 3° results in practically no totally empty grid cells (the histograms in red and blue are practically the same; a tiny difference can be observed in the respective VUE.log files), demonstrating that low-resolution Fourier coefficients giving the overall shape can be recovered nearly completely.

**Figure 8:**
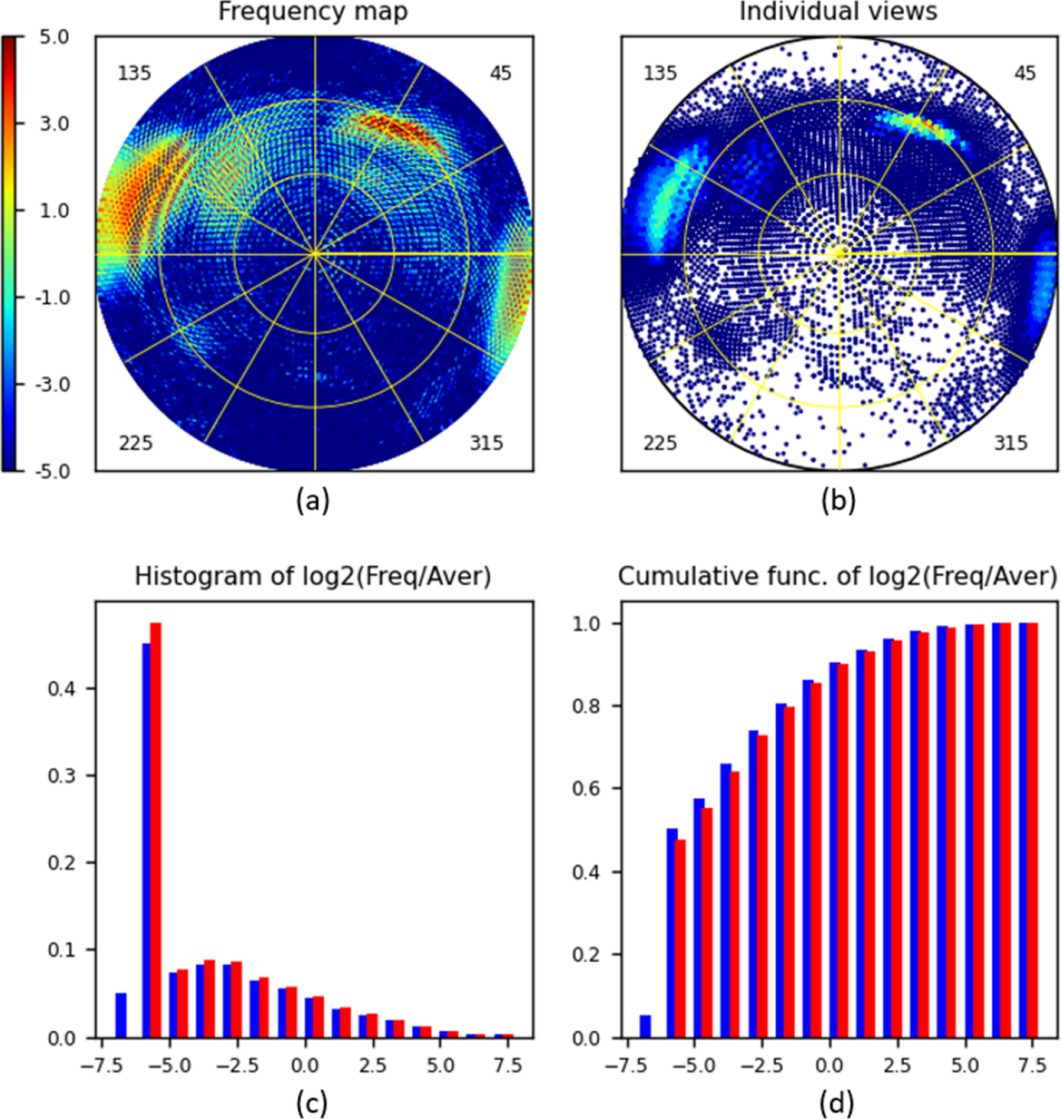
*VUE* diagrams calculated for data set Set3 (about 337 000 projections). The type of diagram and the parameter values are the same as in Fig. 4 except that *d*_max_ = 1°.

As expected, calculations repeated with *d*_max_ = 1° make the distribution map [Fig. 8 ▸(*a*)] grainier. The histogram in blue [Fig. 8 ▸(*c*)] now indicates that about 5% of grid cells are empty and 45% are nearly empty; thus about half of the views are missed or severely under-represented in total. However, these cases are different. For nearly empty cells, some information is present and can be amplified with an appropriate weighting, *e.g.* as discussed above (work in progress). The totally missed views may lead to missed respective Fourier coefficients, especially medium- and higher-resolution ones. Ultimately, one can try to restore them computationally. Such structure factor reconstructions for missed cones and planes of reciprocal space have been used in crystallography [see *e.g.* Lunin (1988 ▸)], suggesting similar algorithms for cryo-EM density modification (Terwilliger *et al.*, 2020 ▸).

## 4. Software

### 4.1. Program overview

The algorithms discussed above have been realized in the stand-alone program *VUE* (views on uniform grids for cryo electron microscopy) written in Python3. The program requires a file of parameters with the standard name VUE.dat and a file with the parameters of the projections. The name of this file is defined in VUE.dat and this is the only mandatory parameter. A large list of optional parameters allows the user to adjust the program routinely to their goals and taste, and to the features of the data set and of the project. The program creates up to four diagrams, a text file VUE.log and, by request, an output file with the modified list of views.

### 4.2. Input file of projections

The format of the file of projection descriptions is either identified by its extension or imposed by the input parameters. The default format of the projections file is star. The program *VUE*, being composed of small local modules, can be easily adapted to read files in other formats. As a template, a module for the binary hed files from the software *Imagic* (van Heel *et al.*, 1996 ▸) is included.

Introducing a new file format also requires providing a convention used to describe the projection orientations, *i.e.* how to express the rotation matrix using the given parameters. The article by Urzhumtseva & Urzhumtsev (2019 ▸) and the respective software may help.

### 4.3. Plotted maps and histograms

The program can plot both the view frequency distribution on a hemisphere, presenting it as a map in the Lambert projection, and the distribution of individual views, also in the same projection. Both diagrams are optional and can be selected by the input data of the program; these data can also define the parameters of the diagrams. The direction of the Lambert projection, which by default follows the **OZ** axis, can be changed to any other defined by its spherical angles, *e.g.* after being estimated using the superimposable coarse reference grid.

The histograms are also optional. As mentioned previously, their argument values are taken on a logarithmic scale in base two.

### 4.4. Numeric information

When running, the program traces its progress on the command-line screen.

The output text file VUE.log mirrors the program parameters, especially their modified values and the values of the view histogram, and contains principal statistical information about the data.

If required, the program creates an output file in the same format as the input file. The records of the input file, describing the views, may be completed by the frequency of a given view and the weight calculated according to the chosen scheme. These records can also be either randomly removed, in the case of their over-representation, or multiplied according to the chosen scheme, so that the output file contains the views distributed more uniformly than initially.

For test goals and a rapid check of the set of views by a subsequent express 3D reconstruction, the program can randomly and uniformly select a given part, as defined by the user.

### 4.5. Program parameters

The program is highly adaptable to individual preferences, both for calculation and for the results presentation. The full list of optional parameters, with the default and allowed values and comments, is given in the file VUE_example.dat. This includes the choice of the type of diagrams to be prepared, the grid type and its size, the frequency calculation parameters, the weighting and colour schemes, the colour bar size, the dot size for individual views, the symmetry operators, the parameters of the eventual view set correction, and many others.

Some auxiliary parameters, for example those defining the position and size of labels on the plots, titles *etc.*, cannot be modified using keywords but are accessible and commented at the top of the Python script and therefore can be modified from there.

### 4.6. System and software requirements

The program uses the standard Python3 (Version 3.7.4 was used to develop the program) with its libraries *NumPy* (Version 1.18.4 or higher; Harris *et al.*, 2020 ▸) and *MatPlotLib* (Version 3.4.3 or higher; Hunter, 2007 ▸), while some older versions may work as well (not tested).

Calculation and plotting of the frequency map with the number of projections of the order of 10^5^ to 10^6^ and with the grid number *M* = 90 recommended for accurate illustrations takes about a minute or less on an ordinary laptop computer. When the number of views approaches 10^6^, drawing the map of individual views becomes the most time-consuming step (this time is independent of the grid size). However, as discussed above, representing this distribution for so large a number of views may not be a good choice.

The program, the file VUE_example.dat and some test data are available on request from the authors or from the web sites https://git.cbi.igbmc.fr/sacha/vue-cryo-em-software and https://ibmc.cnrs.fr/en/laboratoire/arn-en/presentation/structures-software-and-websites/.

## 5. Discussion

Uneven view distributions may deform reconstructed images and therefore complicate their interpretation with atomic models. A number of cases can be found in the literature [for example Sorzano *et al.* (2021 ▸), and references therein]. This makes analysis of the distribution of views in cryo-EM, and the subsequent eventual correction of such sets, an important issue. Several existing computer tools, previously used in other research fields, can be successfully adapted to such analysis. They include uniform grids on discs, the Lambert azimuthal equal-area projection and the kernel density estimation.

The developed program *VUE* gives a practical example of such frequency analysis using these tools. This program, available as stand-alone Python-based software, can be easily adapted to the features of different projects and data. It can be routinely integrated into other packages as a whole or by its components.

The output program information, in both numeric and graphic formats, can be used for a qualitative analysis of the data and indication of eventual problems, as well as for illustrations in publications. A more advanced goal is to use these data to improve the image distortions and artefacts caused by correcting the distribution of views. The program proposes several options for such correction. Comparison of weighting schemes and details of processes to correct the sets of views are separate projects beyond this program description.

As a further development, the suggested method of fre­quency calculation could be extended by choosing an individual σ value in (4) for each view and allowing consideration of a different accuracy of the views. Also, different weighting and view-correcting schemes can be suggested and implemented.

## Appendix A Technical details of uniform grids

Following Roşca (2010 ▸), one constructs *M* concentric equidistanced circles inside a disc of radius *R*, which in our case is equal to  ; the radius  of a circle  is therefore  . The arc  of each circle *m* is divided into *m* equal parts with their bounds φ_*mk*_ =  ,  . The lines connecting the points with indices  are nearly straight lines. With the circular arcs, they form ‘near quadrangles’ for  . The grid in other sectors,  , is built by mirror symmetry about the coordinate axes and the diagonals. The cells on the four diagonals are ‘sectors’ where the diagonals themselves are internal points [Fig. 3 ▸(*b*)].

This uniform Roşca grid on a hemisphere contains  cells in total. These grid cells have surface areas nearly equal to each other and to  , with the difference reducing to zero when *M* increases. We refer to a grid cell by the integer indices (*m* ≥ 1; *k* ≥ 1) of its outer corner (largest values of *m* and *k* over all its vertices). Thus, the cells around the origin, with the outer circle *m* = 1, are denoted by (1; 1), (1; 2), (1; 3) and (1; 4). The indices (*M*; 1) characterize the most distant cell following the **OX** axis. Its three neighbours have indices (*M* − 1; 1), (*M*; 2) and (*M*; 8*M*). The neighbouring cell with respect to the fourth side should have indices (*M* + 1; 1); however, this cell belongs to the south hemisphere. Instead, we consider its equivalent in the north hemisphere, *i.e.* the cell with the indices (*M*; 4*M* + 1). The cells on the diagonal closest to the **OX** axis, *i.e.* with  , have indices (*m*; *m* + 1) and not (*m*; *m*), and similarly for the other diagonals.

This grid recalls a grid built in Cartesian axes with easy identification of the neighbouring cells. Its disadvantage is a particular treatment of the cells on diagonals.

The grids constructed according to Masset *et al.* (2011 ▸), with the inner circle *m* = 1 divided into *N* equal parts (*N* is an extra parameter), do not have singular cells, except those inside this inner circle. Each next circular layer  contains *N*(2*m* − 1) equal cells. Their total number is  and their surface area is  , exactly the same for all of them. The cells are indexed in the same way as described above, by  . Calculating the indices of such cells is slightly easier than for the Roşca grids. On the other hand, it is less trivial to identify neighbouring cells. This step may be required when calculating the distribution map and when identifying the frequency of individual views.
